# Biomarker Analysis of Stored Blood Products: Emphasis on Pre-Analytical Issues

**DOI:** 10.3390/ijms11114601

**Published:** 2010-11-17

**Authors:** Julien Delobel, Olivier Rubin, Michel Prudent, David Crettaz, Jean-Daniel Tissot, Niels Lion

**Affiliations:** 1 Service Régional Vaudois de Transfusion Sanguine, route de la Corniche 2, CH-1066 Epalinges, Switzerland; E-Mails: julien.delobel@mavietonsang.ch (J.D.); olivier.rubin@mavietonsang.ch (O.R.); michel.prudent@mavietonsang.ch (M.P.); david.crettaz@mavietonsang.ch (D.C.); niels.lion@mavietonsang.ch (N.L.); 2 Faculté de Biologie et Médecine, Université de Lausanne, rue du Bugnon 21, CH-1011 Lausanne, Switzerland

**Keywords:** labile blood products, aging and storage lesions, biomarkers, pre-analytics, proteomics

## Abstract

Millions of blood products are transfused every year; many lives are thus directly concerned by transfusion. The three main labile blood products used in transfusion are erythrocyte concentrates, platelet concentrates and fresh frozen plasma. Each of these products has to be stored according to its particular components. However, during storage, modifications or degradation of those components may occur, and are known as storage lesions. Thus, biomarker discovery of *in vivo* blood aging as well as *in vitro* labile blood products storage lesions is of high interest for the transfusion medicine community. Pre-analytical issues are of major importance in analyzing the various blood products during storage conditions as well as according to various protocols that are currently used in blood banks for their preparations. This paper will review key elements that have to be taken into account in the context of proteomic-based biomarker discovery applied to blood banking.

## 1. Blood Components Preparation and Storage

Whole blood transfusion is nowadays realized in very limited cases. Indeed, transfusion therapy now mainly relies on administration of the blood component really needed by the patients. The three main labile blood components used in transfusion therapy are erythrocyte concentrates (ECs), platelet concentrates (PCs) and fresh frozen plasma (FFP). These products can be obtained either by processing a whole blood donation (450–500 mL), which is the most common and simplest technique, or by apheresis where only the needed component is taken from the donor, the remainder been returned to the donor [1]. This last technique is more expensive and requires a more consistent facility.

The standard procedure of blood products preparation from whole blood donation is as follows: once collected in plastic bags containing citrate phosphate dextrose (CPD) anticoagulant, whole blood is centrifuged in order to separate blood cells according to their size and density. Red blood cells (RBCs) settle, while plasma remains on the top. White blood cells and platelets (PLTs) form a “buffy coat” layer at the interface. Finally, the three components are distributed among the sterile inter-connected blood bags by applying a semi-automated pressure to the centrifuged bag containing the original whole blood donation.

In order to prevent various post-transfusion reactions and anti-HLA allo-immunisation, whole blood donation is impoverished in white cells by filtration (erythrocyte plasticity allows to pass through the membranes, contrary to white cells that are retained). Indeed, as leukocytes could be pathogens containing cells, blood products are systematically leukodepleted. A retrospective study has demonstrated that the rates of febrile nonhemolytic transfusion reactions (FNHTRs) are decreased by a factor of 1.7 and 4.1 after RBCs and PCs transfusion respectively, since the establishment of systematic prestorage leukodepletion [2].

Each component obtained from whole blood has optimal storage conditions, which permits to preserve its specific activities and functions. The temperature is a particularly important storage parameter regarding the viability and the quality of products intended for transfusion. Supplemented with an additive solution, generally a saline-adenine-glucose-mannitol (SAGM) solution, RBCs can be stored for up to 42 days from +2 °C to +6 °C, in order to preserve the functionalities of erythrocytes. On the contrary, PLT are stored from +20 °C to +24 °C up to 5 days, with sufficient agitation to permit a good oxygenation and to prevent platelet aggregation. Storage at room temperature promotes bacterial proliferation, and thus increases the risk of transmitted bacteria. The dilemma is that if platelets are transfused after refrigeration at 4 °C, they are rapidly cleared from the recipient circulation [3]. Finally, as indicated by its name, FFP has to be frozen, for an optimal storage, at least at −25 °C, for up to 36 months.

These limits depend on local legislation, and depend on the additive solutions used in erythrocyte and platelet concentrates. These three labile blood products can be further differentially processed depending on demand for particular biomedical needs (γ-irradiation, washing and so on).

## 2. Blood Products Storage Lesions

Even under current optimal storage conditions, modifications and/or degradation of blood components occur in blood bags. These alterations, known as “storage lesions”, affect lifespan and quality of the stored blood products [4]. Even though it is unknown if storage lesions are consequences of the natural aging of blood components, these lesions are well described in the literature.

### 2.1. Erythrocyte Concentrates Storage Lesions

Red blood cells storage lesions can be classified in different categories, depending on their physical or chemical properties [5,6].

First, some biochemical changes related to the energy metabolism occur. It appears that components such as ATP, which is necessary for multiple cellular processes, and 2,3-DPG, which plays an important role in oxygen release, rapidly decrease during the storage. ATP level is considerably diminished after 5 weeks of storage while the 2,3-DPG is almost null after 2 weeks of storage [7]. The low concentration of 2,3-DPG increases hemoglobin affinity for oxygen, which cannot be delivered anymore. However, these levels are rapidly recovered in blood circulation after transfusion of the erythrocyte concentrate. It is also known that intracellular sodium and potassium levels are altered through storage. Indeed, Na^+^/K^+^ pumps are inactive at 4 °C, thus allowing high sodium influx and potassium loss [7].

Then, RBCs storage induces biomechanical changes. The erythrocytes rheological properties, such as shape, deformability, aggregability, and intracellular viscosity are altered during storage [8]. All these changes impact the red blood cells ability to pass through microvessels, thus altering their oxygenation capacities. This is very problematic in blood banking, and neither SAGM nor phosphate-adenine-glucose-guanosin-saline-mannitol (PAGGSM), the two mostly used additive solutions, are described to prevent these storage lesions [9].

Finally, modifications also take place at the protein level. Indeed, erythrocytes are subjected to oxidative lesions, which result in protein oxidative modifications [10,11], hemichrome formation and Band 3 clustering. Storage-induced protein degradation appears to be greatly reduced when oxygen is removed and blood is stored under helium [12]. Antonelou *et al.* have shown that erythrocyte proteins are less oxidized when RBCs are stored in CPD-SAGM compared to storage in CPD-Adenine [13]. Mechanisms of oxidative damage along the development of storage lesions and investigations of the effect of blood anaerobic storage conditions have been recently described by Yoshida and Shevkoplyas [14].

Some aspects of RBC aging occurring *in vivo* and *in vitro* during storage are similar. For example, the increase of intracellular calcium level induces microvesiculation and externalization of negatively-charged membrane phospholipids (phosphatidylserine) [15]. RBCs clearance from blood circulation is thought to be immunologically mediated. Several studies have led to the so-called Band 3 clustering model [16,17]. Oxidized hemoglobin compounds aggregate to form hemichromes that accumulate at the inner side of erythrocytes membrane, covalently bound with cytoskeletal proteins such as spectrin, thus inducing alterations of RBC deformability. Interaction of hemichromes with cytoplasmic domains of Band 3 leads to Band 3 clustering. This conformational change of the major erythrocyte membrane protein is recognized by naturally occurring anti-Band 3 auto-antibodies (nAbs) [18–20]. Paleari *et al.* have demonstrated that the number of these RBC-bound IgGs increases in old population enriched fractions (through Percoll density fractionation), as well as in some selected clinical cases of patients with altered RBC survival [21]. These observations lead to the conclusion that RBC-bound IgG is a biomarker of aging. Hemicromes, Band 3 dimerization and erythrocyte recognition by nAbs are thus additional RBCs aging markers that can be investigated.

### 2.2. Platelet Concentrates Storage Lesions

Platelet Storage Lesions (PSL) consist in morphological changes, platelet activation, platelet proteolysis and platelet surface receptor expression [22,23]. Changes of platelet membrane glycoproteins are also reported in numerous papers [24–26].

The normal platelet discoid shape (also referred as resting shape) is found to be lost after 5 to 7 days of storage at 22 °C. At this storage time, mainly spherical or fragmented platelets remain. Granule release and platelet activation occur during PLT storage, as indicated by the accumulation of *β*-thromboglobulin and platelet factor 4 in the storage medium, and the increase in surface levels of P-selectin (CD62P), respectively.

*In vitro*, loss of aggregation functionality is also observed. There is a significantly storage-dependant decrease of platelet aggregation response to a number of agonists used alone, such as adenosine diphosphate (ADP), epinephrine, collagen and arachidonic acid [27]. However, these aggregation agonists seem to have a synergistic action since pairs of them have been shown to restore platelet aggregation, even after five days of storage [27]. Shapira *et al.* have also shown that platelet prothrombinase activity and membrane phosphatidylserine exposure are enhanced during PCs blood banking storage [28].

As blood components natural aging and storage lesions can directly affect cells, and also have implications at the protein level, the analysis of biomarkers of such phenomenon must be ruled out by important pre-analytical considerations.

## 3. Analysis of Blood Products Aging and Storage Lesions Biomarkers: Pre-Analytical Issues

### 3.1. Importance of Pre-Analytics in Biomarker Discovery Field

In order to be used in medicine, a biomarker has to be both sensitive and specific. Indeed, a biomarker of a given physiological state must allow the identification of this state (sensitivity) and must not be relevant to another physiological state (specificity). However, only few of them meet all criteria making an efficient biomarker suitable for clinical uses.

In the field of biomarker discovery, an important issue is to control sample preparation steps and pre-analytical factors. Specificities of a biomarker have to be the same everywhere following the same protocol. For that reason, the standardization of procedure controlling pre-analytical steps (sampling, conservation and preparation) has to be elaborated.

As discussed before, the three main labile products are differentially processed and stored in order to maintain their viability and quality for transfusion therapy. Standards are published by competent authorities in each country. However, even if the procedure is similar, there are still minor changes applied worldwide. Indeed, blood labile products have to meet definite standards, whatever the way of production and the material used. For example, quality of platelet concentrates varies according to the platelet additive solution used [29].

### 3.2. Proteomics in Blood Transfusion

#### 3.2.1. Proteomic Tools for Biomarker Discovery

Proteomics has been largely used in the field of biomarker discovery [30,31] as well as in transfusion medicine [32,33] since proteins are the effective form of genes, and are thus more able to inform about the physiological state. Indeed, contrary to the genome, the proteome composition fluctuates during time, changes among cell population and is physiological state-dependent.

Clinical proteomics is a major asset for diagnosis, prognosis or evolutionary biomarkers. Varieties of improved proteomic technologies allow separation, identification and quantification of proteins, here potential biomarkers. Gel-based technologies (two-dimensional gel electrophoresis, 2DE, and two-dimensional differential gel electrophoresis, 2D-DIGE), as well as chromatographic techniques (e.g., reverse phase liquid chromatography, RPLC, and strong cation exchange liquid chromatography, SCX-LC) have been developed in order to fractionate proteins or peptides from complex samples. Then, proteins can be analyzed by mass spectrometry (MS) consisting in the ionization of proteins or peptides in order to identify them according to their mass-to-charge ratio (*m*/*z*). The two commonly used sources of ionization are matrix-assisted laser desorption/ionization (MALDI) and electrospray ionization (ESI). MALDI source is preferentially combined with time-of-flight (TOF) analyzer, whereas the ESI source is coupled with quadrupole (Q), ion trap (IT), fourier transform ion cyclotron resonance (FTICR), Orbitrap or combinations of these analyzers. Peptide mass fingerprinting (PMF) or *de novo* sequencing by tandem mass spectrometry (MS/MS or MS^2^), combined with powerful bioinformatic tools allow protein identification by database interrogation. Protein quantitation [34,35] can be achieved thanks to diverse treatments before or after the sampling step [36] (metabolic stable isotope labeling of amino acids in cell culture, SILAC [37], and chemical addition of isotope-coded affinity tags, ICAT [38], or isobaric tags for relative and absolute quantitation, iTRAQ [39]), by spiking samples before MS analysis with known amount of an isotopically labeled analyte (absolute quantitation of proteins, AQUA [40]) and even by recently developed label-free methods, based on MS or chromatography data-processing (spectral counting [41] or total ion current, TIC, integration [42]).

Surface enhanced lazer desorption/ionization time-of-flight (SELDI-TOF), also known as ProteinChip Array technology, is widely used in biomarker discovery [43,44]. A solid-phase chromatographic surface allows sample decomplexification according to chemical or biochemical properties. Users can design their own chromatographic surface to target specific protein population. Bound proteins are then ionized and analyzed as in classical MALDI-TOF MS analysis. However, SELDI-TOF MS analysis does not lead to protein identification, but only gives a mass list of proteins showing relative abundance differences between samples. Once targeted, these proteins have to be further purified then identified by standard mass spectrometry. Caputo *et al.* have developed such kind of methods for protein identification on ProteinChip surfaces, *i.e.*, on-chip digestion and MS/MS sequencing by hybrid quadrupole-TOF [45].

Recent advances in all these proteomic techniques have improved reproducibility, but the problem of inter-laboratory reproducibility still subsists. To ensure this reproducibility, a control of the initial state of samples, with standardization of sample collection, storage and preparation conditions, is required. According to Lehmann *et al.* [46], the following pre-analytical steps have to be controlled: the sampling procedure and the type of container, the temperature and the time before transport (if transport needed), the time between sample collection and sample processing, the process itself (centrifugation, aliquoting, type of secondary container), the storage (temperature and duration) and the pre-treatment just before proteomic analysis.

The general complexity of a proteome, and particularly the huge dynamic range of protein abundance composing the blood proteome, makes necessary the fractionation of blood samples before proteomic analysis, by depletion or fractionation processes. Then, other sample preparation can be required depending on the type of analysis to be performed, and cannot be standardized to all biomarker discovery studies.

#### 3.2.2. Involvement of Proteomics in Blood Transfusion

##### 3.2.2.1. Erythrocyte Proteome Investigations

Red blood cell is an abundant and easily obtained biological material having the particularity to be nucleus- and organelle-free. These characteristics have made of it a model of choice for membrane studies. Nowadays, thanks to development of proteomic tools and technologies, erythrocyte proteome begins to be well documented. Several major studies about RBC proteome have been published last decade, showing a great improvement in terms of number of identified proteins, in cytosolic as well as membrane extracts.

In 2002, a first study combining one dimensional (sodium dodecyl sulphate polyacrylamide gel electrophoresis, SDS-PAGE) and 2D electrophoresis, allows identification of 84 unique proteins from ghost preparation by MALDI-TOF MS analysis. Classic SDS-PAGE reveals 25 proteins of high hydrophobicity and high molecular weight, undetectable with 2DE [47]. Two years later, the use of LC-MS has enabled Goodman and coworkers to identify 181 proteins after tryptic digestion of both cytoplasmic and membrane preparations [48]. Then, Pasini *et al.* have increased the number of identified proteins to 566 (314 membrane-associated proteins and 252 cytoplasmic proteins), combining high-accuracy and high-sensitivity protein identification technologies to analyze trypsin-digested differentially extracted proteins from cytoplasmic and membrane fractions. They have tested different extraction methods leading to differences in number of identified proteins. This number also appeared to vary according to interrogated database [49]. In 2008, the peptide ligand library technology [50] made possible the exploration of red blood cell hidden proteome by reducing the erythrocyte dynamic protein concentration range, which permits Roux-Dalvai *et al.* to identify 1578 soluble proteins by nano-LC-LTQ-Orbitrap MS/MS analysis [51]. A more recent study reveals 222 identified proteins. Even though this number may appear low in comparison with other studies, the authors have set up an interesting hemoglobin depletion strategy, derived from the existing HemogloBind™ reagent [52]. In 2010, van Gestel *et al.* have explored RBC membrane proteome combining blue native protein separation (BN/SDS-PAGE), CyDyes labeling-based quantitation and LC-MS/MS identification [53]. This allowed them to identify 524 proteins, of which 155 are membrane proteins. The CyDyes labeling led to quantitation of a 40% decrease in spectrin levels in erythrocyte membrane fraction of a patient presenting RBC membrane disorder. This interesting approach thus seems to be applicable to biomarker discovery. Lately, efforts have been made in the establishment of interactome and network maps, as reported by Goodman *et al.* [54] and updated by D’Alessandro *et al.* [55].

##### 3.2.2.2. Platelet Proteomic Analyses

Platelet quality was usually determined from measurable characteristics such as pH, platelet morphology and hypotonic shock responses. Protein analysis of platelets during storage was first achieved in the end of the 1980s by identifying variation in actin [56,57]. However, actin polymerization is linked to the method used in the preparation of PCs and it has been shown that this process is partially reversible after 1 day of storage [58], which is highlighting the implication of pre-analytics in biomarker discovery. Since the introduction of proteomics, new tools have been brought in order to characterize platelet storage lesions. As explained previously, lesions occur during storage of PCs and can alter several proteins and mechanisms. The proteome analysis of platelets during the period of storage may reveal different biomarkers, potentially important for improving platelet quality [59]. Proteomic analysis of platelets *in vivo* or during storage was achieved at the beginning of the last decade [59,60]. Using gel-based proteomics, different groups identified several hundreds to thousands of PLT proteins [61–63]. Based on peptidic-centric methods, 641 proteins were reported by Gevaert and coworkers, including hydrophobic membrane proteins [64]. Later, different studies have focused on changes during storage periods, typically between a 1-day and a 7-day storage. Thiele *et al.* have shown by DIGE and MS that 97% of cytosolic proteins do not change over a 9-day storage period [65]. However, in the 3 remaining percent, they highlighted that, for instance, spetin 2 and gelsolin show changes in 2DE. They claimed that these proteins, affected during apoptosis, may be suitable markers for platelet alteration during storage. Using a similar approach but focusing on supernatant, it has been also shown that levels of proteins increase during storage, which may have some implications for transfusion recipients [66]. More recently, Devine and coworkers have used complementary proteomic methods (2DE, DIGE, iTRAQ and ICAT) to investigate protein changes between days 1 and 7 of PLT storage [67]. Hence, 503 proteins changed concentration was reported thus completing the PLT proteome.

Beyond these analyses, the impact of such potentially biomarkers has to be integrated within molecular mechanisms leading to platelet storage. As explained by Schubert and Devine, biological interpretation of proteomic data has to be taken into account [59]. A comprehensive view of different mechanisms related to biomarkers may be required to understand how to improve platelet quality. Even though such an approach is far to be completed nowadays, changes in molecular mechanisms have been described. Schubert *et al.* have identified 12 proteins connected in one potential signaling pathway underlying storage lesion development [68]. They have shown that PI3-kinase-dependent Rap1 activation leads to integrin αIIbβ3 activation and PLT degranulation. In addition, a PI3-kinase inhibitor incubated with PLT for 7 days plays a role in Rap1 activation and seems to improve PLT integrity and quality during storage. A global view, including protein-protein interactions [69], is one of the tools able to provide a wide picture of the storage lesions and mechanisms involved during such modifications.

All these studies have potential influence on the future of platelet quality and safety during storage, and of course, as it is explained within this review, pre-analytic considerations are inherent to these studies.

### 3.3. Blood Proteomics Pre-Analytical Considerations

All these blood-related studies can be impacted by pre-analytics. From sample collection to proteomic analysis, all manipulation steps are source of pre-analytical variation. The main pre-analytical considerations to take into account are resumed in Table 1.

#### 3.3.1. Sample Collection

Blood donation conditions such as the donor position, the time of tourniquet application, and the needle bore size are important. In upright position, blood macromolecular component rates can be increased, due to a shift in body water from intravascular to interstitial compartment (macromolecules cannot pass through blood vessels and are thus concentrated) [70]. The same effect can be noticed if the tourniquet application exceeds one minute. Indeed, proteins and macromolecules can thus accumulate upstream the tourniquet and be detected at a higher rate than normal by this effect of concentration. Moreover, Cengiz *et al.* have shown in a recent study that erythrocyte deformability is still altered up to 180 seconds after tourniquet removal, and erythrocyte aggregation is increased up to 30 seconds after tourniquet removal [71]. They also confirmed that tourniquet application induces leukocytes activation.

Concerning the needle bore size, too thin needle can induce hemolysis, thus free hemoglobin and other protein concentrations increase in serum/plasma. It is not problematic when working with blood products obtained from whole blood donation because in case of large blood drawn volume, largest needles are used (16 to 19-gauge needles). In routine venipuncture, smaller blood volumes are drawn and patient comfort is of high importance, thus thinner needles are chosen (21 to 23-gauge needles). However, too thin needles (25-gauge needles) should be avoided since it can induce pre-analytical variability (hemolysis), leading to false interpretation of test results [72,73].

#### 3.3.2. Sample Container Type

All containers have to be tested for a possible interference with analyses to be performed. Indeed, containers can salt out plastic components, which can be detected in analysis by mass spectrometry. The presence of polymers in an MS analysis induces effect of signal suppression: the high amount of ionized polymer saturates the detector, and the other sample minority components ions cannot be detected anymore. In 2004, Drake *et al.* have demonstrated the presence of polymers on mass spectra from extracts of saline solution incubated in different blood sample containers, from two different manufacturers [74].

Investigating serum/plasma proteomes, Hsieh *et al.* have shown that there was no significant difference between samples collected in glass red-top tubes and samples collected in serum separator tiger-top tubes [75]. However, comparing the nature of the anticoagulant used, they have found high significant differences in plasma protein profiles. Most common anticoagulants are EDTA, heparin and sodium citrate. Their different action ways lead to these differences in proteome composition. Moreover, they are also known to interfere with subsequent analysis. EDTA is known to interfere in enzymatic activity assays, whereas heparin is not advised for some ELISA-based assay kit.

#### 3.3.3. Sample Processing and Handling

First of all, the time between blood donation and preparation of the labile products is of high importance. Banks *et al.* have shown in 2005 that this duration induces differences in SELDI-TOF profiles of plasma collected with different anticoagulants and serum samples [76].

A study of West-Nielson *et al.*, based on principal component analysis (PCA) of MALDI-TOF spectra, reveals that there was no significant difference between spectra of serum from blood left to clot up to 24 h at 4 °C and from blood left to clot up to 4 h at 24 °C [77]. However, when blood is left to clot for longer times at 24 °C (8 h to 24 h), clearly changes appear in mass spectra, which was confirmed later [75]. In 2007, Timms *et al.* have shown that both transport time and temperature play a role on SELDI-TOF spectra profiles of sera [78].

Studying RBC microparticles, Rubin *et al.* have encountered some pre-analytical caveats. Indeed, our group has shown that sample processing temperature and vortexing duration affect microparticles counting [79]. Dealing with centrifugation force (1500 or 3000 × *g)* and duration (15 *vs.* 30 minutes), serum proteome profiles do not appear to be affected [75].

Protein degradations, such as fragmentation by proteolysis, and protein modification, such as oxidation, have been described as storage lesions (see part 2). However, these alterations can be induced during sample manipulation, and cannot be differentiated from storage lesions. The addition of protease inhibitors to prevent handling-related proteolysis is thus of high importance. If protease inhibitors are omitted, it has been shown that low molecular weight compounds appear and accumulate during sample preparation [80]. It is necessary to prevent proteolytic cleavages because generated peptides could thus be mistaken for potential biomarker. Indeed, analyzing peptidome variations of pathological sera, Davis *et al.* have shown that modifications in low molecular weight compounds reveal disease-induced hemostatic dysregulation rather than direct protease activity of a given pathology [81]. Indirectly pathologically-induced peptidome variations are not specific enough to be considered as good biomarkers and have thus to be avoided by use of protease inhibitors, even if it is not known whether all protease activities are repressed.

RBCs possess a set of enzymes responsible for antioxidant defense (catalase, superoxide dismutase, peroxiredoxins and glutathione peroxidase). However, these enzymes can be inactivated by excess of reactive oxygen species (ROS), and additional protein oxidation can thus occur during sample processing. In order to prevent this phenomenon, particularly prior to cystein oxidation assessment in stored RBC redox proteomic studies, cell-permeable cystein-specific reagents, such as iodoacetamide (IAA) or *N*-ethylmaleimide (NEM) alkylating agents, are commonly used [82,83].

#### 3.3.4. Sample Storage

Storage conditions are important to preserve quality of labile blood products and to ensure the efficiency of transfusion therapy. Thus, it is known that erythrocyte concentrates are preferentially stored at 4 °C whereas platelet concentrates are stored at 22 °C to prevent cold activation of platelets. Finally, fresh frozen plasma is stored at −25 °C at least.

Thus, whole blood storage cannot ensure optimal preservation of each blood components. In labile blood products aging field, samples are ECs, PCs or FFPs, and are stored according to blood banking legislation. In pathological-case biomarker research, samples are either serum or plasma, and are not subject to any storage legislation. Several studies aimed at investigating the reproducibility of analysis after storage at −20/−30 °C or −80 °C, and after freeze-thaw cycles [75,84,85]. It appears that storage at −20 °C instead of the recommended −80 °C storage does not induce loss of information. Samples stored at −20/−30 °C can thus be used for biomarker discovery [86].

Some proteins have been shown to be highly temperature-sensitive: cryoproteins. Described for the first time in the 1930’s by Wintrobe [87] as myeloma’s patient sera precipitating at cold temperature, cryoproteins are serum or plasma proteins having the property to precipitate below the physiological temperature. Most of the time composed of immunoglobulins (mainly IgM) these precipitates are called cryoglobulins (CG), and are classified under three categories according to the universally adopted classification of Brouet [88]. Other proteins have this particularity to precipitate at low temperature, as cryofibrinogen (CF) (for instance, see the study on clinical cases of patients with either CF alone or combined CF plus CG [89]) and cold agglutinins. Proteomic techniques are well adapted to determine the protein composition of cryoprecipitates [90]. Cryoproteins are potential biomarkers, and pre-analytical precautions have to be taken for their investigation. Indeed, blood must be sampled in anticoagulant-free, 37 °C pre-heated container, handled and centrifuged at 37 °C, in order to prevent cryoprotein elimination by precipitation before analysis.

## 4. Conclusions

Pre-analytics is of very high importance for all kind of test, assay and discovery field, and more importantly in biomarker discovery field, due to the direct clinical implications. Uncontrolled pre-analytical parameters may lead to false interpretation of results.

In clinical proteomics, reproducibility is indispensable and is straightly correlated with pre-analytical considerations. Standardization of sample harvesting, process, and storage has to be set up in order to minimize pre-analytical variations. Biomarker discovery is such a complicated field that there is no time to waste in wondering if an observed protein variation is due to different physiological states or simply to differences in sample handling or storage. Many small-scale standardized protocols exist in laboratories and lots of pre-analytical parameters have been investigated so far. Time is now about setting up worldwide-scale standardization of sample handling, process and storage.

The human proteome organization (HUPO) has set up the plasma proteome project (HPPP), in order to perform a comprehensive analysis of plasma and serum protein constituents in people. A specimen collection and handling committee (SCHC) was created to evaluate pre-analytical variables that can potentially impact experiment results. In this purpose, Rai *et al.* have conducted in 2005 a proteomic study, comparing serum and plasma analyses, evaluating storage and handling conditions as well as the use of protease inhibitors [91]. They were thus able to present general recommendations helping researchers to set up more robust plasma proteome studies. This is an important step towards the establishment of standardized protocols for proteome studies, which is primordial in the biomarker discovery field.

This kind of initiative is important for the transfusion medicine community and for the whole clinical medicine community as well. Reviews about importance of the pre-analytics in many fields are regularly published nowadays, which means that procedures standardization is a real contemporary thought.

## Figures and Tables

**Table 1.** Main pre-analytical conditions to respect for proteomic analyses of labile blood products and blood samples in biomarker discovery field.

			**Labile blood products**			**Blood sample for pathological-case biomarker research**	**References**
			**ECs**	**PCs**	**FFP**		
Sampling	sample obtention	donor position	resting position				[68]
		tourniquet application	less than 30 seconds if possible, no more than 1 minute				[69]
		needle bore size	avoid too thin needle (21–23 gauge needles are preferred)				[70,71]
	container type	material	approved plastic bags			vacuette®-like blood collection tubes	[72,73]

		anticoagulant content	Citrate Phosphate Dextrose (CPD)			depends on analyses	[73]

processing and handling	time between sampling and processing		up to 24 h at +4 °C or up to 4 h at +24 °C				[74,75]
	transport time and temperature		controlled transport at +4 °C, must be as brief as possible				[76]
	centrifugation		prefer centrifugation at +4 °C, up to 3000 × g				[73]
	use of protease inhibitors		required to avoid proteolysis				[78,79]
	use of antioxidant reagents		needed in case of redox proteomic studies				[80,81]
storage	temperature	original sample	+4 °C	+22 °C	−25 °C	−20/−30 °C or −80 °C	[73,82,83]

		proteinic extracts	−20/−30 °C and −80 °C stored extracts are both suitable				[84]
	freeze/thaw cycles		better only once, but several cycles do not affect proteomic patterns				[73,82,83]
	case of cryoproteins		always work at 37 °C (pre-heated containers)				[87]
